# Palpation. Pus and Proteid

**Published:** 1907-07

**Authors:** 


### PALPATION, PUS AND PROTEID.

Since the medical profession has become more proficient in the diagnosis of diseases of the prostrate gland, the greater part of the obscurity, which so long was more closely associated with sexual disorders than any other class of diseases, has been and is being rapidly cleared away. Chronic or recurrent gonorrhœa can often be promptly cured if the condition of the prostate and seminal vesicles be borne in mind and appropriate treatment instituted.

Conservative authorities now admit that 75 per cent. or 80 per cent. of chronic urethral discharges are caused by or associated with a prostatitis or a vesiculitis. It is because this source of irritating toxins and reinfections is not understood that so many men are under the impression that gonorrhœa can never be entirely cured. Urethral strictures are widely known as a cause of gleet but it seems that the medical profession, as whole, is very slow in realizing the importance of carefully examining the prostate and vesicles as a routine in all persistent or obscure sexual and urinary disorders.

The acute inflammation and the hypertrophy occurring in old men can readily be diagnosed by palpation and by the history and are much less frequently overlooked than are the chronic diseases of mild character which as a rule have no symptoms pointing directly to the prostate nor is there usually a history of an acute prostatis. There are three cardinal points to be remembered in these examinations and which enable one to make an accurate diagnosis, they are palpation, pus and proteid. A mistaken idea is prevalent that by palpation alone an accurate diagnosis can be made. This may be true generally, as there is more/or less irregularity and enlargement or a nodular condition in chronic prostatitis, but this change is frequently so slight that unless the examiner has had considerable experience an error is easily made if this test alone is depended upon.

The second point is to examine a fresh or stained specimen of the secretion from the prostate by massage and appearing at the meatus or the sediment of the urine passed to wash it out. Of course the urethra and bladder must be eliminated as possible sources of pus by irritating them with a physiologic salt solution when the second glass of urine is not clear. Pus cells in the secretion from the prostate always indicate that the gland is inflamed whether it be enlarged or not.

The presence of proteid in the urine after massage of the prostate when none is found before, also means that there is prostatitis and is a useful test when a microscope is not available. A satisfactory method of making the test is to superimpose urine upon a saturated solution of magnesium sulphate 10 parts to which has been added 1 part of nitric acid, as a layer test. This will clearly demonstrate the presence of any proteid by a white ring at the zone of contact. If the urine be albuminous before massage an irrigation of physiologic salt solution should be given and a few ounces of it left in the bladder to be passed out after the secretion from the prostate has been expressed. This fluid is then tested for proteid.

---

The Report of the Commissioner of Education for the year ending June 30th, 1905, has just been received and shows many interesting facts indicating the advances made in education in the United States. During the year 1904-5 there were 18,887,846 pupils enrolled in the institutions comprising our educational system, being an increase of 263,585, as compared with the previous year. The total sum expended for all the education was more than one-half the cost of the National Government.

---

The Atlanta School of Medicine last winter essayed something new in this part of the South by opening and operating two hospital wards for teaching purposes. At first considered in the nature of an experiment, the hospital has developed into a very strong teaching factor, and the authorities now announce that plans have been perfected for running it permanently in a thoroughly systematic manner.

The advantages of actual bedside teaching are inestimable, and we believe such advances, wherever made, will receive a hearty response from the profession.

Without disparaging the merits of any other college, it may truthfully be said that this one has achieved surprising success through its energetic efforts toward the betterment of medical education.

